# Immune endotypes in tuberculosis: Keys to decoding disease complexity

**DOI:** 10.1111/joim.70092

**Published:** 2026-04-03

**Authors:** Shamila D. Alipoor, Julia Guthrie, Lina Davies Forsman, Andrew R. Di Nardo, Susanna Brighenti

**Affiliations:** ^1^ Division of Medical Microbiology and Molecular Medicine Department of Clinical and Experimental Medicine Linköping University Linköping Sweden; ^2^ Ludwig Boltzmann Institute for Network Medicine at the University of Vienna Vienna Austria; ^3^ Division of Infectious Diseases Karolinska University Hospital Stockholm Sweden; ^4^ Department of Medicine Solna Karolinska Institutet Solna Sweden; ^5^ Global Tuberculosis Program Department of Pediatrics Baylor College of Medicine Houston Texas USA; ^6^ Department of Internal Medicine and Radboud Center for Infectious Diseases Radboud UMC Nijmegen the Netherlands; ^7^ Center for Infectious Medicine (CIM) Department of Medicine Huddinge Karolinska Institutet, ANA Futura Huddinge Sweden

**Keywords:** clinical phenotypes, endotypes, host‐directed therapy (HDT), immune response, *M. tuberculosis*, tuberculosis

## Abstract

Tuberculosis (TB) remains a major global health challenge, with multi‐drug antibiotic regimens as the current standard of care. While effective at killing *Mycobacterium tuberculosis*, these treatments do not resolve persistent inflammation, prevent lung damage, or reverse immune dysregulation that contribute to poor outcomes and disease recurrence. Precision medicine offers a promising alternative but requires deeper insight into disease mechanisms to enable tailored interventions. This comprehensive review introduces the concept of immune endotyping to define the underlying disease mechanisms as tools to decode clinical and immunological heterogeneity in TB. TB displays a wide spectrum of clinical phenotypes, from latent or asymptomatic infection to mild or severe disease with characteristic non‐cavitary or cavitary lung pathology. Instead, distinct immune endotypes capture the diverse biological pathways that shape disease progression and treatment response. Similar clinical presentations may arise from different immune dysfunctions, underscoring the need to move beyond broad phenotypic classifications. Advances in multi‐omics and computational analyses uncover immune signatures that enable stratification for host‐directed therapies (HDTs) targeting hyperinflammation, immunosuppression, coagulopathy or metabolic exhaustion. Integrating clinical, radiological, and immunological data through multimodal profiling is essential for developing personalized interventions. We also explore how endotyping has transformed treatment in other diseases, offering valuable insights for TB. Additionally, we present examples of how putative immune endotypes may be targeted with appropriate HDTs. In summary, this review underscores the potential of immune endotypes to advance precision medicine in TB, moving beyond one‐size‐fits‐all treatment to improve outcomes, especially in severe and drug‐resistant cases.

## Graphical abstract

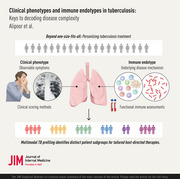

## Introduction

Every 20 s, tuberculosis (TB) claims a life, making it one of the most devastating infectious diseases worldwide. TB is an intracellular bacterial infection requiring daily treatment with multiple antibiotics for 6–9 months. The prolonged and complex antibiotic treatment contributes to the emergence of drug resistance in *Mycobacterium tuberculosis* (Mtb), fueling the global spread of multidrug‐resistant TB (MDR‐TB) that requires even longer regimens [1]. Antibiotics effectively eliminate the mycobacteria, but fail to resolve the chronic inflammation that drives persistent lung damage and cardiovascular disease, and do not reverse the immunological hyporesponsiveness linked to post‐TB cancers and recurrent infection [2]. In addition to discovery of novel therapeutic targets in Mtb, host‐directed therapies (HDTs)—aiming to modulate the host immune response through various mechanisms—represent a promising yet underexplored strategy for improving treatment outcomes, particularly in patients with more severe forms of TB. Although TB is typically diagnosed and treated using a one‐size‐fits‐all approach, tailored therapies require identifying the clinical profile of TB along with immune endotypes, defined by distinct molecular traits or pathophysiological mechanisms that shape disease progression.

In this review, we outline how immune endotyping can uncover underlying disease mechanisms and provide a powerful framework for addressing the biological heterogeneity of pulmonary TB. TB is a multifactorial disease with wide variation in symptoms and radiological severity, reflecting underlying differences in host immune pathways that are likely to give rise to distinct immune endotypes. Advances in immune endotyping allow a deeper exploration of how host responses vary across TB presentations, which is an essential step toward effective implementation of HDTs. Most clinical studies have compared immune responses in latent TB infection to active TB disease and healthy controls. Although useful for diagnostics and vaccines, advancing treatment requires similar analyses within active TB subgroups. Particularly, understanding immunological differences between mild, moderate, and severe TB cases with and without cavitation could yield critical insights.

Identification of distinct TB endotypes may enable more accurate diagnostics and personalized treatment strategies, particularly for drug‐resistant or atypical TB cases. As seen in oncology, autoimmune diseases and other immune‐mediated diseases, integrating clinical phenotyping and immune endotyping in TB paves the way for effective stratified medicine approaches that account for individual variabilities in disease expression and immune function. Here, we also provide examples illustrating how patient stratification based on immune endotypes can further accelerate the development and implementation of tailored HDTs for distinct patient subgroups.

### Immune responses in TB and their role in disease progression

**Fig. 1:**
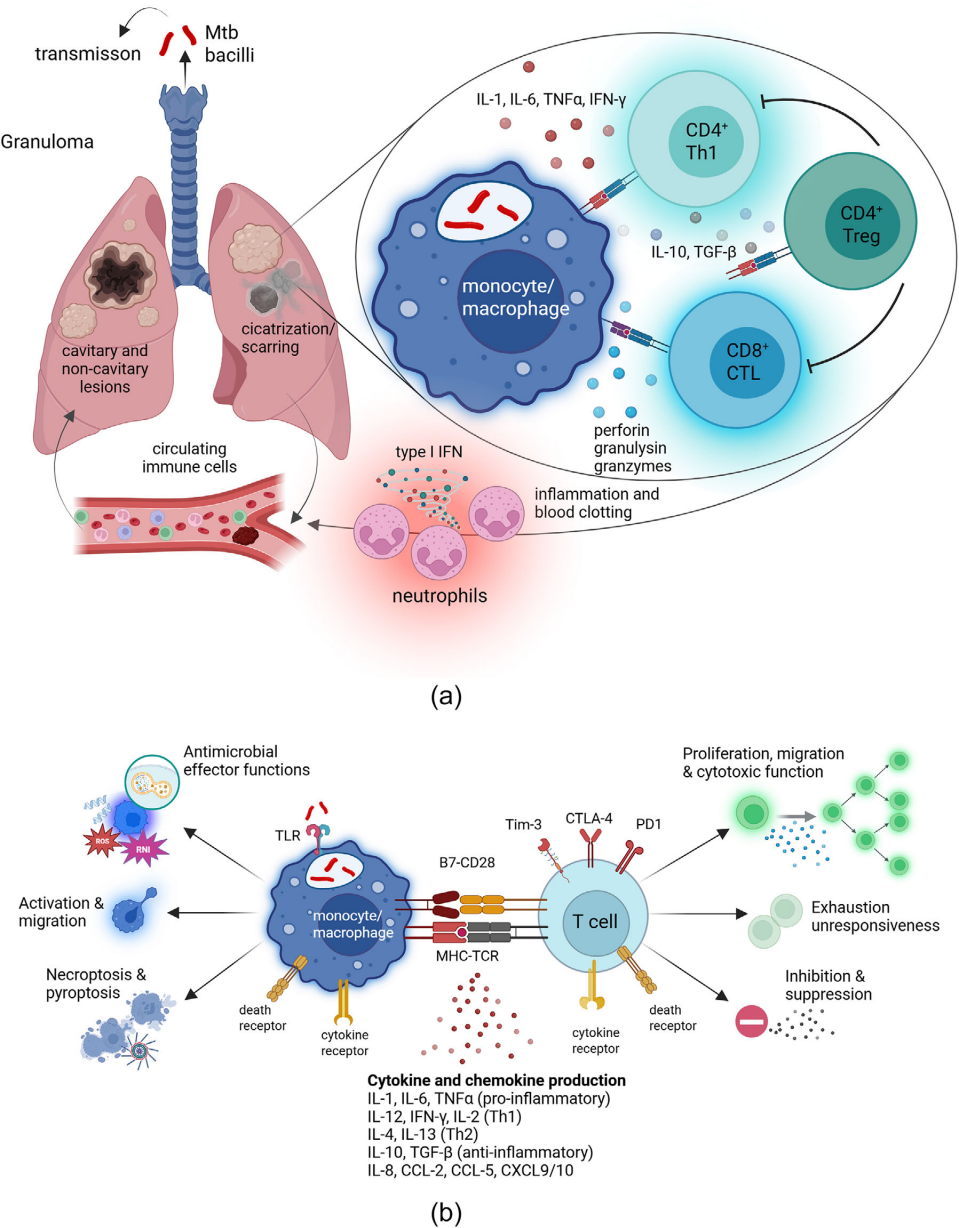
Immune cell interactions and functional signaling in tuberculosis (TB). This figure illustrates the complex immune landscape of Mycobacterium tuberculosis (Mtb) infection in the lungs. (a) The schematic highlights key anatomical and cellular components involved in TB immunity, including granuloma formation, cicatrization and sterilization, or progression to non‐cavitary and cavitary lesions. It depicts the roles of monocytes/macrophages, neutrophils, CD4+ and CD8+ T cells, and regulatory T cells (Tregs), which migrate between the site of infection in the lung and the peripheral circulation. Pro‐inflammatory cytokines, such as IL‐1β, IL‐6, and TNF, drive macrophage and T cell activation, including the induction of cytotoxic T cells that produce effector molecules like perforin, granulysin, and granzymes. Systemic inflammation activates coagulation pathways and thrombosis, contributing to caseous necrosis and cavitation. Expansion and activation of Tregs help dampen excessive inflammation, potentially through the production of anti‐inflammatory cytokines IL‐10 and TGF‐β, downregulation of the high‐affinity IL‐2Rα (CD25) limiting cytokines responsiveness and proliferation, downregulation of co‐stimulatory molecules CD80 and CD86 (B7‐1 and B7‐2), signaling via inhibitory receptors PD‐1 or CTLA‐4, or receptor‐mediated killing of effector T cells via death‐receptors such as Fas‐FasL or TRAIL/DR5 pathway. See (b) for details. (b) The second panel details receptor signaling pathways and functional responses of macrophages and T cells during Mtb infection. Signal 1 (antigen‐specific activation via MHCI or II and T cell receptor [TCR]) and Signal 2 (costimulation via CD28 and B7) initiate immune cell activation and cytokine production. Macrophages respond by generating reactive nitrogen and oxygen species (reactive nitrogen intermediates [RNI] and reactive oxygen species [ROS]), antimicrobial peptides such as cathelicidin, and inducing autophagy to enhance intracellular Mtb clearance. Cell death receptor signaling (Fas‐FasL, TNFR1, TRAIL) and inflammasome activation trigger apoptosis, necroptosis, or pyroptosis. Inhibitory pathways via Tim‐3, PD‐1, and CTLA‐4 contribute to T cell exhaustion and unresponsiveness. Both macrophages and T cells express diverse cytokine receptors that mediate stimulatory and inhibitory signals. Together, the panels underscore the delicate balance between protective immunity and immune‐mediated pathology in TB. IFN, interferon.

Pulmonary TB accounts for 80%–85% of cases and is the most infectious form, whereas extrapulmonary TB represents 15%–20% and involves organs outside the lungs, mainly lymph nodes, pleura, abdomen, or bones [209]. Although TB is fundamentally an infectious disease, it also displays key features of an immunological disorder. Mtb hides within alveolar macrophages and has evolved sophisticated mechanisms to evade host defenses by altering inflammation, metabolism, and tissue remodeling at the site of infection [3]. Cellular immunity is central in TB control, with myeloid cells including monocytes and macrophages serving as primary host cells and lymphocytes acting as main effector subsets [4, 5] (Fig. 1a). Pro‐inflammatory cytokines activate macrophages and dendritic cells, triggering antimicrobial pathways and promoting antigen presentation to prime Th1 and cytotoxic T cell responses [6] (Fig. 1b). Although the immune response in TB has been extensively reviewed elsewhere [3, 4, 6], we briefly highlight the diversity of selected immune pathologies and their potential links to clinical manifestations in TB.

#### Granulomatous lesions in TB: containment, collapse, and clinical consequences

The pathological hallmark of human TB is the formation of granulomas, which are organized immune cell aggregates centered around Mtb‐infected macrophages, surrounded by T cells and enclosed within a fibrotic capsule [7]. Granulomas aim to contain infection but can also serve as niches for bacterial persistence and dissemination [8]. Th1 cytokines, particularly IFN‐γ and TNF‐α, are critical for immune cell activation, recruitment, and granuloma organization at sites of infection [9]. TNF‐α supports early granuloma formation and cooperates in synergy with IFN‐γ to stimulate macrophages and enhance intracellular elimination of Mtb [9, 10]. Chemokines, such as CCL2, IL‐8, CCL5, CXCL9 and 10, further orchestrate the coordinated recruitment of myeloid and effector T cells required for granuloma formation and bacterial control [11, 12]. Protective immunity also involves cytolytic and antimicrobial effector molecules such as perforin, granzymes, and granulysin that target infected cells and mediate direct killing of Mtb bacilli via osmotic lysis [13]. However, excessive TNF‐α and IFN‐γ can promote chemokine overproduction, leading to overt immune cell infiltration, macrophage necroptosis, caseous necrosis, and tissue damage [14, 15, 16]. Th2 cytokines such as IL‐4 and IL‐13 also influence granulomatous responses and tissue remodeling in TB by counterbalancing strong Th1‐driven inflammation [17, 18, 19].

Granulomas are dynamic and heterogeneous structures that mirror tumors in their chronic antigen exposure, sustained inflammation, and progression to immune exhaustion or suppression [20]. Excessive inflammation fuels lung pathology and cavitation—accelerating disease progression—whereas weak or insufficient effector T cell responses permit bacterial replication and dissemination [5, 20]. Over 70 years ago, George Canetti analyzed more than 1500 autopsies and described the dynamic nature of granulomas, observing that some can undergo calcification and cicatrization, a process almost always associated with mycobacterial sterilization [21] (Fig. 1a). In contrast, caseous granulomas may soften and evolve into cavities, allowing infectious liquefied material to spread bacteria through the airways [21, 22] (Fig. 1a). These early insights suggested that distinct immune endotypes in TB may lead to divergent pulmonary pathologies and clinical outcomes.

Cavitary TB has been linked to exaggerated inflammation driven by neutrophils and macrophages producing excess matrix metalloproteinases (MMPs) [23, 24]. Although prolonged inflammation contributes to tissue destruction [25], Th2‐dominant responses may also promote collagen deposition, fibrosis and tissue remodeling. In contrast, non‐cavitary TB with limited pathology often shows stronger Th1 immunity, aiding bacterial containment [26]. Moreover, a lack of CD4+ and CD8+ T cells at cavity surfaces may impair contact‐dependent killing of infected cells [27], whereas cytokine‐unresponsive T cells within granulomas also fail to control bacterial growth [28]. Systemic dissemination of Mtb—called miliary TB—as well as isolated hilar lymphadenopathy is more common in individuals with deficient Th1 responses [29], supporting the idea that distinct immunopathological mechanisms underlie diverse radiological manifestations in TB.

#### Inflammatory imbalances in TB: platelet and neutrophil‐driven pathology

TB exhibits physiological and immunological features that are shared with several other chronic inflammatory conditions. Accordingly, persistent inflammation in TB is characterized by excessive release of pro‐inflammatory cytokines such as IL‐1, IL‐6, and TNF‐α, alongside a range of other inflammatory mediators [3]. Systemic inflammation in TB can promote endothelial damage, enhance coagulation, and suppress fibrinolysis [30, 31], thereby contributing to clinical complications such as venous thromboembolism [32]. Pulmonary TB has been shown to induce a systemic hypercoagulable state, characterized by increased activation of coagulation mediators and simultaneous impairment of anticoagulant pathways [33]. Pro‐inflammatory IL‐6 and other cytokines stimulate the coagulation cascade while interfering with anticoagulant mechanisms, resulting in an increased tendency for blood clot formation, which can worsen TB prognosis (Fig. 1a). Thrombocytosis, defined as a heightened platelet count, is a common manifestation of systemic inflammation and frequently observed in patients with pulmonary TB [34]. Although IL‐6 appears to be required in the early phase of infection to activate macrophages and restrict Mtb growth [35], high IL‐6 levels are usually considered detrimental to immune control and clinical TB outcomes [36, 37].

Another widely recognized marker of acute inflammation and tissue damage is an elevated neutrophil count [38], and growing evidence underscores the critical contribution of neutrophil function in shaping disease outcomes in TB [23]. Accordingly, monitoring neutrophil dynamics has emerged as an important tool for assessing immune responses and gauging the severity of pulmonary pathology in TB [23]. Active TB has been shown to elicit a neutrophil‐driven Type I interferon (IFN) response, which antagonizes the protective IFN‐γ‐mediated immunity and contributes to necrosis and caseation within granulomatous lesions [39, 40]. Consistently, transcriptional profiles reflecting Type I IFN signaling and neutrophilic inflammation have been found to correlate with disease severity in murine models of TB [40, 41, 42, 43], revealing potential targets for HDTs.

#### Dysregulated immune responses in TB: balancing protection and pathology

The immune system must eliminate intracellular Mtb while limiting tissue‐damaging inflammation, yet the regulatory mechanisms that maintain this balance remain poorly defined. Innate immune cells serve as the first line of defense by recognizing and engulfing Mtb, but the pathogen can also exploit these cells to establish infection and evade clearance [44]. Notably, neither IFN‐γ‐producing CD4+ Th1 cells nor cytotoxic CD8+ T cells employing granule‐mediated killing are sufficient to eradicate infection [45]. Instead, Mtb subverts host defenses by creating hyperinflammatory yet immunosuppressive microenvironments characterized by hypoxia, necrosis, fibrosis, and localized immune suppression, all of which collectively impair effective immune clearance [20].

TB pathogenesis is further shaped by dysfunctional T cell subsets—including exhausted, pathogenic, and regulatory T cells (Tregs)—that suppress protective immunity and worsen disease severity [46, 47, 48] (Fig. 1b). In parallel, dysregulated myeloid populations—particularly the expansion of myeloid‐derived suppressor cells (MDSCs)—further dampen host defenses [49, 50]. Lipid‐rich mycobacteria promote metabolic reprogramming in MDSCs from glycolysis to fatty acid oxidation (FAO), enhancing their suppressive functions and potentially supplying nutrients to Mtb [51]. Additionally, the activation of anti‐inflammatory pathways, including IL‐10 and TGF‐β, helps limit tissue damage but also suppresses protective responses, enabling bacterial persistence [45].

Persistent antigen stimulation in chronic TB leads to the upregulation of inhibitory receptors such as PD‐1, TIM‐3, and CTLA‐4 on Mtb‐specific T cells [52, 53], which help limit immune‐mediated pathology but may also prevent the proper activation of effector T cells [54] (Fig. 1b). PD‐1 blockade in Mtb‐infected non‐human primates exacerbates inflammation, disrupts T cell function and trafficking into granulomas, and ultimately increases bacterial burden [55]. Similarly, cancer patients with latent TB who receive PD‐1 inhibitors as part of immune checkpoint therapy face an elevated risk of reactivating active TB disease [56]. Although PD‐1 can restrain harmful inflammation, it also marks exhausted T cells with impaired metabolic activity and antimicrobial capacity that fail to control infection effectively [52, 53, 57]. At the opposite end of the immune spectrum, excessive TNF‐α signaling promotes pathological inflammation, yet TNF‐α inhibition in rheumatoid arthritis patients with latent TB greatly increases the risk of reactivation due to weakened effector T cell responses [58]. Together, these findings illustrate the complexity of immune regulation in TB and highlight the need to better understand how immune heterogeneity shapes disease progression and outcomes.

### Clinical diversity of TB disease with relevance for immune endotypes

**Fig. 2:**
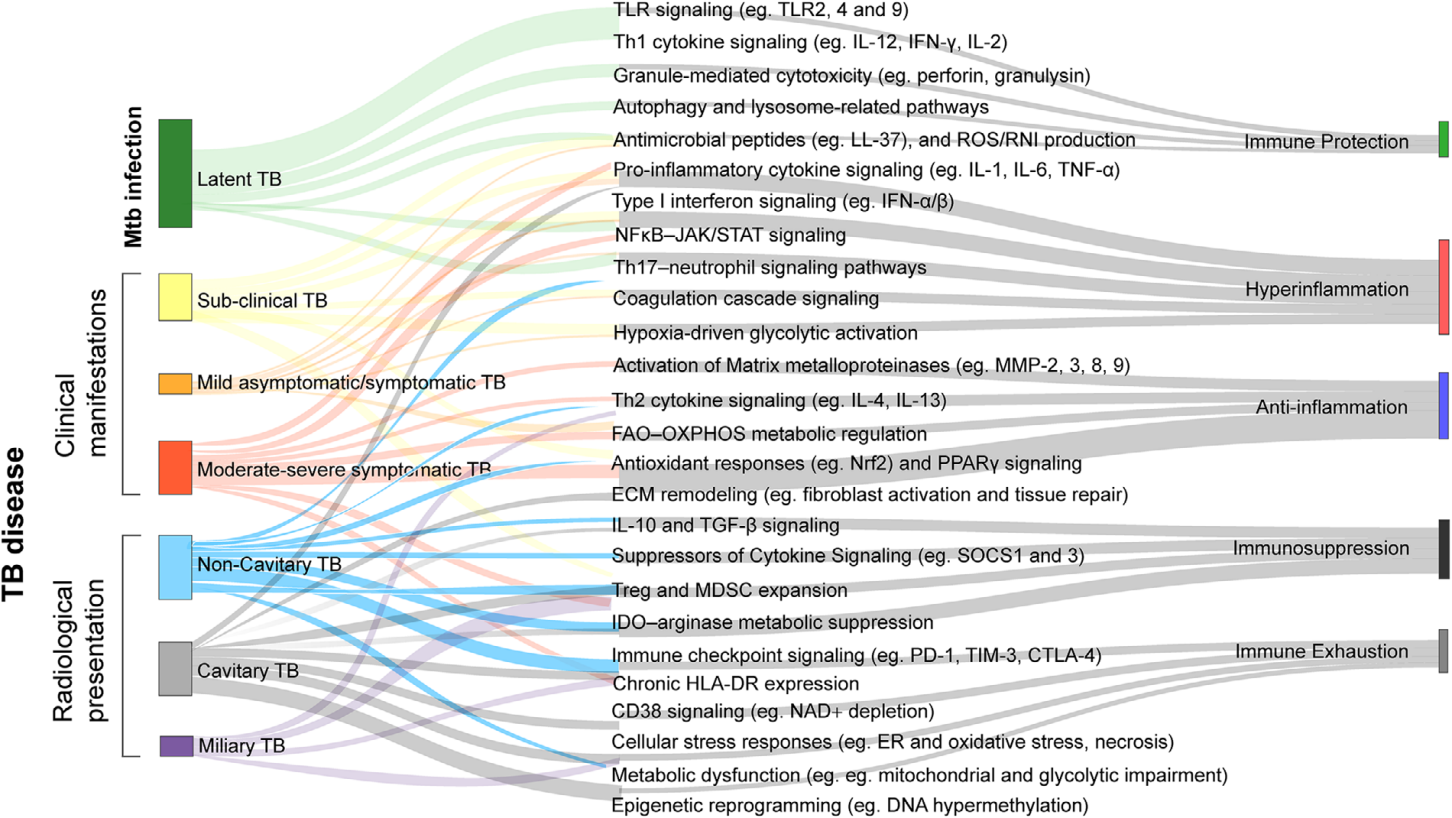
Mapping clinical tuberculosis (TB) phenotypes to diverse immune endotypes. This Sankey diagram schematically illustrates the branching relationships between clinical TB phenotypes and their associations with putative immune endotypes. Radiological and symptomatic presentations, including latent TB, subclinical TB, mild/asymptomatic TB, moderate‐to‐severe symptomatic TB, non‐cavitary TB, and cavitary TB, may be linked to distinct yet overlapping immune signaling pathways. These immune endotypes reflect underlying biological processes such as protective immunity (e.g., Toll‐like receptor [TLR] signaling, cytotoxic lymphocyte activation, autophagy), hyperinflammation (e.g., Type I interferon [IFN] and neutrophil activation, coagulation cascade signaling), anti‐inflammatory responses (e.g., PPARγ signaling, fatty acid oxidation and oxidative phosphorylation [FAO–OXPHOS] metabolic shift), immune suppression (e.g., SOCS signaling, FOXP3 activation), and immune exhaustion (e.g., immune checkpoint signaling, CD38 activation, epigenetic reprogramming). The size and proportion of the colored boxes representing clinical TB forms and immune endotypes are intended for visualization purposes only. The diagram underscores how similar clinical manifestations may arise from distinct immune mechanisms, highlighting the importance of integrated profiling to guide personalized treatment strategies and host‐directed therapies.

The basis for precision medicine in TB is to move beyond the one‐size‐fits‐all approach toward individualized treatment strategies tailored to patient subgroups with distinct immune endotypes, each reflecting specific pathophysiological mechanisms of disease (Fig. 2). Endotypes are defined by molecular and functional traits, including immunological, metabolic, transcriptional, and/or genetic properties [59, 60], and are further influenced by comorbidities such as HIV or helminth infections, diabetes, host–microbiome interactions, and bacterial virulence factors. In contrast, clinical phenotypes refer to observable manifestations of illness including symptoms, physical findings, imaging characteristics, and biochemical markers [61]. TB encompasses a broad clinical spectrum, ranging from asymptomatic infection to mild or severe, and sometimes disseminated, life‐threatening disease. Systematic clinical phenotyping is essential for capturing this diversity, and for understanding how these presentations are shaped by radiological features and heterogenous host immune responses.

We propose that diverse clinical phenotypes of TB represent interconnected stages along a continuum of immune, metabolic, and tissue‐remodeling responses, spanning from effective immune containment to widespread pathology, severe tissue damage, and immune exhaustion (Fig. 2). In the following section, we outline each clinical phenotype depicted in Fig. 2 and discuss their hypothetical relationships with the immune pathways and regulatory mechanisms described above.

#### Latent TB infection

In most exposed individuals, TB infection enters a latent state in which Mtb is effectively contained without clinical symptoms or radiological abnormalities. During latency, bacterial replication is balanced by host immunity, often for many years. This state is thought to reflect robust protective responses, including Toll‐like receptor (TLR) signaling, especially TLR2, and strong Th1 cytokine production (IL‐12, IFN‐γ, IL‐2), but reduced Th2 (IL‐4, IL‐13) signaling [62]. Antimicrobial pathways in macrophages including autophagy and lysosome‐mediated intracellular degradation, production of antimicrobial peptides such as LL‐37 and defensins, and generation of reactive oxygen species and nitrogen intermediates, contribute to direct killing of Mtb [45]. Additionally, granule‐mediated cytotoxic effector molecules—including direct exposure to granulysin—can force Mtb into a dormant state, thereby supporting granuloma stability [63]. The combination of these mechanisms can restrict bacterial growth and limit tissue damage, helping maintain an asymptomatic, contained infection. However, although progression from latent to active TB is relatively infrequent, spontaneous clearance of Mtb is also rare, indicating that even effective immune containment is generally insufficient for bacterial eradication.

#### Subclinical and incipient TB

If the immunological balance is shifted in favor of the pathogen, latent Mtb infection may progress to subclinical or incipient TB [64]. At this stage, immune containment begins to weaken, but overt clinical disease has not yet emerged, although radiological abnormalities may already be detectable [64]. Consistently, low levels of MMPs, such as MMP‐9, may be present in early tissue lesions and contribute to the initial stages of granuloma formation [61, 65]. Bacteria enter a state of slow metabolic activity and replication, being biologically active but not yet causing symptoms. Unlike active TB, sub‐clinical TB is associated with lower levels of systemic inflammation, reflected by lower ESR and C‐reactive protein levels, along with stable CD4+ and CD8+ T cell counts and less pronounced T cell exhaustion [66].

In contrast, early activation of innate immune pathways—particularly Type I/II IFN signaling—together with early suppression of Th17 responses, has been shown to precede progression from infection to active TB [67]. This phase also involves the activation of additional inflammatory pathways—including NF‐κB and JAK/STAT signaling, elevated platelet and coagulation cascades, and hypoxia‐driven glycolysis—indicating heightened immune activity within infected tissues despite absent or minimal symptoms. Overall, subclinical TB represents a pre‐symptomatic, high‐risk stage with a substantial likelihood of progressing to active disease in the absence of intervention.

#### Mild and moderate‐to‐severe pulmonary TB

As disease advances, individuals may develop mild asymptomatic or symptomatic TB, marked by further amplification of inflammatory responses and emerging metabolic reprogramming. Pro‐inflammatory cytokine signaling involving IL‐1, IL‐6, and TNF‐α becomes more pronounced, and counter‐regulatory pathways—including Th2 cytokine signaling and early antioxidant responses mediated by Nrf2 and PPARγ [68]—begin to appear. These regulatory processes help limit excessive inflammation but may also compromise effective antimicrobial responses and bacterial clearance, ultimately promoting Mtb persistence [69].

Active TB is also associated with a neutrophil‐driven Type I IFN signature that disrupts protective immunity by impairing IFN‐γ signaling in macrophages, leading to excessive neutrophil recruitment and failure to control Mtb replication [39]. Enhanced proinflammatory responses, together with reduced IFN‐γ and IL‐17 levels and a shift toward a Th2‐biased cytokine profile, are accompanied by induction of SOCS3 in infected macrophages [18]. We have shown that these immunological alterations are markedly more prevalent in patients with progressive pulmonary TB than in those with localized TB lymphadenitis or pleuritis [18, 70], supporting the notion that these changes reflect features of more severe disease.

Furthermore, patients with reduced cytolytic T cell responses—marked by diminished perforin‐ and granulysin‐producing cells—exhibit less control of Mtb infection [71, 72]. Our observations demonstrated that this defect is particularly evident at the local site of infection, where low frequencies of CD8+ T cells co‐expressing perforin and granulysin within granulomatous lesions correlate with higher bacterial loads, cavitation, and drug‐resistant TB disease [73, 74, 75]. In addition, tissue lesions in active TB display low LL‐37 expression but increased infiltration of FoxP3+ Treg cells, suggestive of impaired anti‐TB effector responses that could unleash bacterial growth and dissemination [74, 75]. Extensive evidence also shows that chronic inflammation in TB drives expansion of Treg subsets [46, 76] as well as MDSCs [49, 77], both of which are associated with progressive, severe TB disease and treatment failure.

In moderate‐to‐severe TB, immune dysregulation escalates and is accompanied by profound tissue remodeling and metabolic adaptation. Persistent inflammation drives extracellular matrix (ECM) breakdown, fibroblast activation, and tissue‐repair responses, reflecting ongoing lung damage [78]. At this stage, a metabolic shift of infected macrophages toward FAO and oxidative phosphorylation (FAO–OXPHOS), together with antioxidant and pro‐repair programs, delineates a compensatory but ultimately insufficient attempt to modulate inflammation [79]. Endothelial dysfunction within TB granulomas compromises vascular integrity, and together with suppressed fibrinolysis, promotes a hypercoagulable state and heightened thrombotic risk due to impaired regulation of coagulation pathways [31]. These changes coexist with progressive tissue pathology, worsening symptoms, and high mycobacterial burden.

Immune exhaustion is a key feature of advanced Mtb infection, arising from chronic antigen exposure and a suppressive cytokine milieu [52, 80]. Subsequently, T cell dysfunction and cytokine unresponsiveness permit bacterial persistence and disease progression, characterized by high co‐expression of activation markers such as CD38 and HLA‐DR [81], together with inhibitory receptors, where combined expression of TIM‐3 and PD‐1 denotes more profound exhaustion [52, 80]. Patients with higher bacillary loads exhibit more pronounced T cell impairment, with MDR‐TB patients showing markedly elevated levels of exhaustion markers such as CTLA‐4, PD‐1, and TIM‐3 compared with drug‐susceptible cases, reflecting the deeper immune exhaustion associated with persistent, difficult‐to‐treat infections [82]. Although detrimental, exhaustion also serves as a regulatory brake to limit immune‐mediated tissue damage during prolonged inflammation.

Using a validated TB symptoms score, we previously sub‐grouped patients into mild, moderate, or severe clinical disease [12, 83, 84]. Higher TB scores strongly correlated with elevated inflammatory markers such as ESR and plasma IL‐6, whereas BMI, hemoglobin [83] and albumin [12] showed inverse correlations. Anemic patients were more often underweight and displayed higher ESR along with reduced CD4/CD8 T cell counts and diminished Mtb‐specific IFN‐γ responses [83]. Mild disease was characterized by mixed Th1/Th2 profiles, while moderate‐severe TB showed predominance of pro‐inflammatory and B cell‐stimulating cytokines (e.g., BAFF/APRIL, LIGHT/TNFSF14, soluble TNFR, IL‐6) and chemokines (e.g., IL‐8, IP‐10, CCL4) [12]. This chemokine profile suggests recruitment of neutrophils and activated T and B cells and/or Treg subsets that could contribute to pathological inflammation rather than effective immune protection [11, 85]. Notably, clinical TB disease severity did not correlate with the radiological extent of lung damage [12], suggesting that symptom‐based phenotypes may reflect distinct inflammatory immune profiles that do not directly mirror underlying pulmonary pathology.

#### Non‐cavitary and cavitary pulmonary TB

Radiological manifestations further distinguish pulmonary TB phenotypes based on highly variable lung involvement—spanning from minimal or absent parenchymal changes to dense infiltrates, fibrosis, and cavitary destruction. Both cavitary and non‐cavitary TB are shaped by the immune pathways described earlier—particularly Th1/Th2 balance, T cell exhaustion or suppression, macrophage effector capacity, and inflammatory cytokine programs—which collectively influence lesion evolution, bacterial burden, and tissue damage. Distinct immune profiles may therefore drive divergent pathological trajectories in these different disease forms.

Non‐cavitary TB is characterized by inflammatory lesions often dominated by contained Th1 responses and concomitant immunosuppressive signaling. In this phenotype, greater radiological involvement correlates with more severe symptoms and lower serum albumin levels [86]. Clinically, non‐cavitary TB often presents with milder symptoms and responds well to therapy, with overall lower relapse risk compared to cavitary [87, 88].

Cavitary TB, in contrast, represents the most severe localized pulmonary phenotype. Cavities, typically thick‐walled lesions in the upper lobes, harbor mycobacteria in diverse metabolic states shaped by hypoxic granuloma microenvironments [21, 87, 89]. Cavitation is associated with profibrotic and Th2 skewed signaling as well as profound cellular dysfunction, including excessive neutrophil activity and upregulation of MMP 1, 3, 8, and 9, which mediate collagen destruction, tissue necrosis, and cavity formation [24, 90]. Our unpublished observations suggest that cavitation is more strongly linked to platelet‐mediated inflammation, whereas overall parenchymal damage—regardless of cavitation—is more related to neutrophil‐driven inflammation. Platelet activation and aggregation within granulomas—compounded by hypoxia‐induced endothelial injury that impairs anticoagulant function—create a strongly prothrombotic milieu closely associated with cavitation [91]. Additional drivers include cellular stress responses (e.g., endoplasmic reticulum [ER] and oxidative stress) that promote apoptosis and necrosis of infected macrophages [92], and epigenetic changes such as DNA hypermethylation of immunity related genes, which reduce Mtb‐specific responsiveness [93].

Cavitary disease correlates with higher bacterial burden [94], consistent with classic autopsy studies showing that bacterial concentrations are greatest inside cavities and substantially lower burden in surrounding inflamed tissue [21]. A selective absence of CD4+ and CD8+ T cells along the cavity surface may further prevent direct T cell–macrophage interactions at the site of infection [27] that contributes to permissive growth of bacteria. Together, these processes drive extensive lung tissue destruction, high bacterial loads, increased transmission risk, and a difficult‐to‐treat disease phenotype strongly associated with treatment failure and relapse [87].

Overall, TB lung pathology reflects a continuum of lesion types—each linked to distinct bacterial phenotypes, immune responses, and clinical outcomes—emphasizing the need for pathology‐ and immune‐guided therapeutic strategies.

#### Miliary TB

Miliary TB is a rare, severe form of disease caused by widespread hematogenous dissemination of Mtb from the primary infection site that is associated with a high mortality risk if left untreated [95]. It involves multiple organs—including the lungs, liver, spleen, bone marrow, and the central nervous system—and occurs most often in individuals with impaired immunity such as young children, older adults, and immunocompromised patients. Immunologically, miliary TB reflects a failure of granuloma containment, typically driven by insufficient Th1 responses and defective macrophage activation, allowing uncontrolled bacillary spread. Clinically, miliary TB presents with non‐specific systemic features such as prolonged fever, weight loss, and night sweats, often delaying diagnosis and contributing to its severe outcomes.

Overall, integrating these clinical and radiological TB phenotypes within a unified immunopathological framework is essential for improving diagnosis, disease staging, and the development of HDTs tailored to specific immune, metabolic, and tissue‐remodeling pathways.

### Dissecting TB disease heterogeneity through immune endotyping

Building on this framework, the next challenge is to understand how these diverse manifestations map onto distinct immune endotypes that can guide precision medicine approaches, enabling the development of HDTs tailored to specific immune pathways [96]. Endotype‐specific HDTs have the potential to enhance antibiotic efficacy, improve clinical outcomes, and reduce long‐term complications. An overview of putative immune endotypes, their key features, and main implications for HDTs is presented in Table 1. These strategies aim to fine‐tune the immune response—suppressing non‐productive inflammation while boosting antimicrobial effector functions—to minimize lung damage and accelerate recovery. In the absence of effective patient stratification, TB treatment relies on broad, uniform regimens and overtreatment to ensure cure, making the goal of shortening therapy without compromising efficacy a global priority. There is growing recognition that stratifying patients according to both pathogen and host determinants is essential for personalizing drug therapy and optimizing treatment duration. Such an approach may incorporate comprehensive drug‐susceptibility testing, therapeutic drug monitoring, and the integration of HDTs selected on the basis of patient‐specific immune endotypes.

**Table 1:** Overview of immune endotypes in tuberculosis (TB): key features and therapeutic implications.

Putative TB immune endotype	Dominant immune features	Key molecular markers	Pathological associations	Main implications for HDTs
Th1/Th17‐protective granulomas	Robust myeloid and cytotoxic T cell, Th1/Th17 responses with controlled inflammation	IFN‐γ, IL‐17, autophagy, LL‐37, cytotoxic effector signatures	Improved bacterial control with relatively preserved lung structure	Support of protective immunity and avoidance of excessive immunosuppression that might impair bacterial clearance
IFN—high (hyperinflammatory)	Exaggerated Type I IFN signaling, neutrophil‐dominated	IFN‐stimulated gene signatures, activated neutrophils	Severe lung pathology, extensive, and destructive lesions	Anti‐inflammatory HDTs to curb IFN‐ and neutrophil‐mediated damage while maintaining antimicrobial function
IFN—low	Blunted Type II IFN responses, relatively weak cellular immunity	Low IFN‐gene signatures, impaired Th1 responses	Mild disease or poor bacterial control, risk of treatment failure	Immune‐enhancing HDTs to boost IFN‐γ and Th1 effector functions and improve bacterial clearance
Platelet‐driven/thrombo‐inflammatory	Heightened platelet activation, platelet–leukocyte aggregates, pro‐coagulant microenvironment	Elevated platelets, P‐selectin, platelet factor‐4 (PF4/CXCL4), thromboxane pathways	Higher thrombosis risk, endothelial dysfunction, cavitation‐related hypercoagulability, and tissue damage	Anti‐thrombo‐inflammatory HDTs (e.g., aspirin, statins), targeting platelet activation, improving endothelial stability, and reducing hypercoagulability
Neutrophil‐driven	Dominant neutrophilia, IL‐17‐associated inflammation	High neutrophil counts, elevated IL‐6, IL‐17 and related chemokines	Severe pulmonary pathology, tissue‐destructive granulocytic infiltrates	Targeting neutrophilic inflammation (e.g., IL‐17/chemokine pathways) to limit immunopathology
Myeloid‐dysregulated	Expanded dysfunctional monocytes/macrophages, high MDSCs, impaired antigen presentation, excessive inflammatory mediator release	MDSC markers (ARG1, S100A8/A9), low HLA‐DR, high IL‐6, neutrophil‐myeloid gene modules	Severe TB, high bacterial burden, immune paralysis with concurrent hyperinflammation, treatment failure	HDTs to restore myeloid function (e.g., reducing MDSCs, IL‐6/STAT3 modulation) and rebalance inflammatory vs. antimicrobial responses
Lymphocyte‐depleted	Reduced circulating and tissue lymphocytes, impaired adaptive immunity	Low CD4/CD8 T cell counts, reduced activation markers and tissue homing	Pronounced clinical symptoms, poor pathogen control and high mortality risk	Restoring Th1 and cytolytic T cell function to enhance antimicrobial defense
Immunoregulatory/exhausted	TGF‐β‐rich, Treg‐dominated environment, exhausted effector cells	TGF‐β, Tregs, IDO1, PD‐L1+ myeloid cells, exhaustion markers	Poor outcomes with impaired bacterial clearance despite limited overt inflammation	Reversal of immune suppression or exhaustion (checkpoint modulation) to restore effective responses
Th2‐skewed granulomas	Th2‐biased immunity with mast cells, plasma cells, stromal enrichment	Th2‐associated cytokines and transcriptional markers	Bacterial persistence within poorly protective granulomas	Targeted HDTs that shift or counteract non‐protective Th2 polarization
Immunometabolic dysregulation	Altered metabolic pathways in immune cells, energetic, and biosynthetic imbalance	Metabolic gene signatures, dysregulated glycolysis, lipid, and mitochondrial pathways	Disease progression, treatment failure, poor long‐term outcomes	Metabolism‐targeted HDTs to normalize immune cell metabolism and enhance host antimicrobial function

Abbreviations: HDT, host‐directed therapy; IFN, interferon; MDSC, myeloid‐derived suppressor cells.

#### The rationale behind immune endotyping in other diseases

Endotype profiling is already well‐established in asthma, diabetes, rheumatoid arthritis, and cancer, where it is clear that patients with similar clinical features often respond differently to the same treatment [59, 60]. Consequently, endotyping through multi‐omics approaches is transforming precision medicine across a range of inflammatory diseases by enabling patient stratification based on underlying molecular mechanisms, an approach the TB research field could adopt to advance HDT treatment. Recent advances in immune profiling and machine learning have enabled the identification of biologically distinct patient subgroups that extend beyond conventional clinical phenotypes [97]. In asthma, endotypes are broadly classified into Type 2–high, mixed, and Type 2–low forms, each driven by distinct inflammatory pathways [98]. Type 2–high asthma, the most prevalent subtype, is marked by eosinophilic inflammation and Th2 cytokines (IL‐4, IL‐5, IL‐13), with biomarkers such as serum IgE, periostin, and FeNO guiding diagnosis and targeted therapy [99]. Biological therapies—including monoclonal antibodies that block key Type 2 cytokines—effectively reduce inflammation, eosinophil counts, and exacerbations, while improving symptoms and lowering the reliance on broad‐spectrum corticosteroids [99]. In contrast, Type 2–low or mixed endotypes often show neutrophilic, IL‐17‐driven inflammation, and poor steroid responsiveness [100]. Emerging strategies targeting IL‐17, CXCR2, and HMGB1/RAGE pathways or upstream inhibition of cytokine signaling through PI3K, JAK, or MyD88, may offer therapeutic benefit for these difficult‐to‐treat patients [100]. This heterogeneity highlights the importance of precision medicine, aligning treatment with underlying immune mechanisms. A similar strategy is likely feasible for TB, enabling tailored immunomodulation rather than relying on broad‐spectrum immunosuppressive drugs.

Endotyping has transformed understanding of complex inflammatory diseases such as asthma, cancer, systemic lupus erythematosus (SLE), bronchiectasis, and sepsis. In SLE, eight molecular subtypes, defined by IFN signaling, plasmablast expansion, and lymphocyte activity, provide far greater sensitivity than conventional biomarkers [101]. Atopic dermatitis shows similar benefits, with endotypes linked to chemokine and cytokine profiles that support more precise disease monitoring. In sepsis, transcriptomic profiling has revealed coagulopathic, inflammatory, and adaptive endotypes with distinct immune signatures and prognostic implications [102]. Similar to what could be expected in patients with TB, coagulopathic and inflammatory endotypes are associated with poor outcomes, whereas adaptive endotypes display stronger lymphocyte activity and improved prognosis [102]. New transcription‐based frameworks now stratify sepsis patients by inflammatory and antimicrobial response pathways to guide personalized therapy [103]. Consensus clustering of sepsis endotypes has further identified myeloid and lymphoid dysregulation patterns, showing that patients with high lymphoid dysfunction benefit from steroids and IL‐1 blockade (anakinra), whereas mortality increased when patients with low lymphoid dysfunction were treated with steroids [104]. Together, these examples illustrate the power of endotyping to uncover hidden biological heterogeneity and inform tailored interventions. As omics and computational technologies continue to evolve, lessons from other fields provide a strong foundation for applying endotype‐based, host‐directed strategies in TB.

#### Identification of immune endotypes in TB

##### Putative TB endotypes

Although substantial literature supports the existence of multiple immune endotypes in TB, direct confirmation remains limited. Multi‐omics, big data, and systems biology have demonstrated a profound complexity and dynamic variability in TB immunity, both in peripheral blood and in samples obtained from the site of infection in the lung. TB patients exhibit variability not only in the intensity of their IFN responses but also across the complement system, metabolic pathways, and other immune processes, suggestive of distinct immune endotypes [105]. Notably, individuals with elevated IFN signaling (IFN‐rich) tend to experience more severe lung pathology compared to those with lower IFN responses (IFN‐low) [105]. Similarly, a higher frequency of CD4+IFN+IL‐17+ cells has been observed in patients with extensive pulmonary lesions [106]. Other studies have shown that severe pulmonary pathology in TB is associated with elevated neutrophil levels, whereas pronounced clinical symptoms correlate with reduced blood lymphocyte counts [107]. In contrast to many reports, Th1‐producing or polyfunctional CD4+ T cells were not linked to disease severity [107]. These findings suggest that distinct TB manifestations are driven by diverse immune mechanisms, largely influenced by leukocyte composition.

**Fig. 3:**
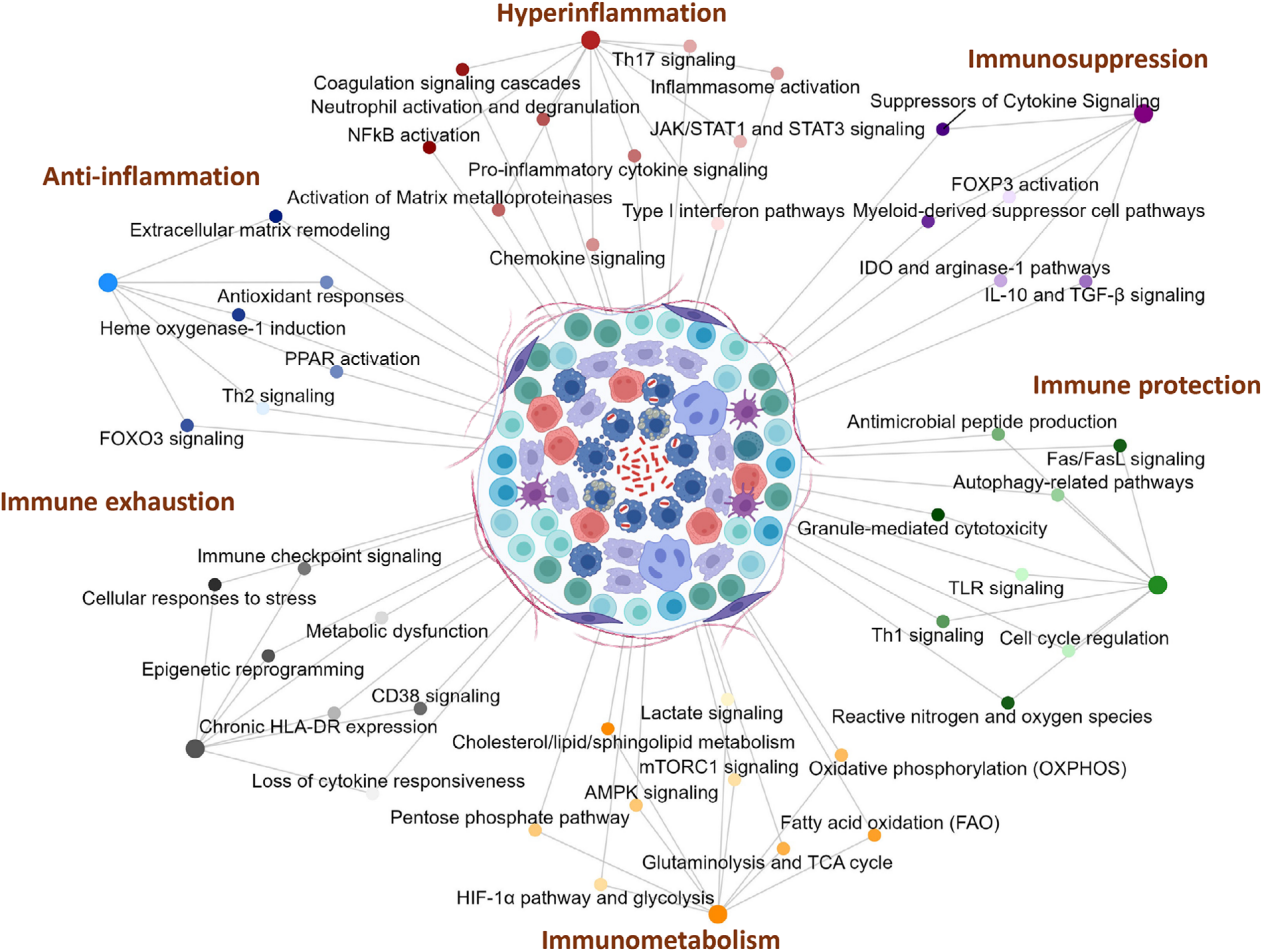
Diverse immune microenvironments within the tuberculosis (TB) granuloma. This network plot schematically depicts the heterogeneous immune microenvironments that emerge within the TB granuloma. Distinct clusters represent spatially and functionally specialized niches shaped by varying immune responses. These include pro‐inflammatory zones dominated by activated macrophages, neutrophils, and Th1 cells producing cytokines such as TNF, IL‐1β, and IFN‐γ; regulatory zones enriched in Treg cells and anti‐inflammatory mediators like IL‐10 and TGF‐β; hypoxic and fibrotic regions associated with tissue remodeling and fibroblast activation; and immunosuppressed areas characterized by myeloid‐derived suppressor cells (MDSCs), checkpoint molecule expression, and metabolic exhaustion. These immune responses may reflect dynamic alterations within individual granulomas over time, whereas a single granuloma can simultaneously contain multiple, functionally distinct zones. The network highlights cellular interactions and signaling pathways that define each microenvironment, reflecting the dynamic balance between bacterial control, immune regulation, and tissue pathology. This spatial complexity underscores the need for localized profiling to inform targeted host‐directed therapies and improve treatment outcomes.

Instead, results from analyses of human TB granulomas have found a vast heterogeneity in the immune‐inflammation profile of individual lesions—which underscores the significant challenges in identifying peripheral blood biomarkers, given the diverse lesion types and the complex, localized immune responses within the lung [20, 21, 108, 109, 110]. TB lesions span a continuum, and host–bacterial interactions within the granulomas are dynamic, shaped by local differences in immune‐signaling pathways that result in divergent trajectories of lesions within the same host (Fig. 3). Advanced spatial imaging has revealed that TB lesions are heterogeneous, consisting of distinct cellular aggregates surrounding necrotic cores [109]. Locally coordinated immunoregulatory programs defined by an IFN‐γ‐depleted microenvironment enriched for TGF‐β, Treg cells, and IDO1+PD‐L1+ myeloid cells were found to correlate with disease severity and outcome [111]. Moreover, Th2 type granulomas enriched for mast and plasma cells along with stromal cells were found to favor bacterial persistence, whereas Th1/Th17 granulomas enriched with cytotoxic T cells were associated with bacterial control [112]. Another study implicated macrophage heterogeneity as a major driver of differential susceptibility to Mtb, providing further insights relevant to future HDT strategies [113]. As illustrated in Fig. 3, these studies suggest the existence of several distinct immune endotypes linked to immune protection, hyperinflammation, anti‐inflammation, immune exhaustion, immunometabolism, and immunosuppression.

##### Defined TB endotypes

To date, the most clearly defined immune endotypes in TB involve inborn errors and deficiencies in IL‐12–IFN‐γ or TNF signaling pathways, which lead to fatal mycobacterial infections in humans [58, 114]. However, the genetic basis of human resistance to Mtb remains largely unknown. A recent review categorized TB patients into Advantageous and Disadvantageous endotypes based on the activation of distinct cellular populations, both myeloid and lymphocytic, and humoral mediators such as cytokines and lipid signaling molecules [115].

Recently, two different immune endotypes—A and B—and their associated clinical outcomes and gene expression profiles were proposed based on retrospective analysis of whole blood transcriptomics obtained from pulmonary TB patients [116]. Accordingly, Endotype A was defined by enhanced inflammation and immunity, whereas Endotype B was associated with enhanced metabolic activity (OXPHOS) and proliferative pathways [116]. Endotype A demonstrated slower bacterial clearance and clinical improvement, and cavitary disease was more common compared to Endotype B [116]. Cell trajectory analysis of transcriptome data suggested an ordered progression from healthy controls to Endotype B and then Endotype A [116], driven by metabolic exhaustion and decreased immune cell proliferation upon chronic antigen stimulation [116]. Whether a branched trajectory includes additional, as‐yet unidentified immune endotypes remains to be determined.

**Fig. 4:**
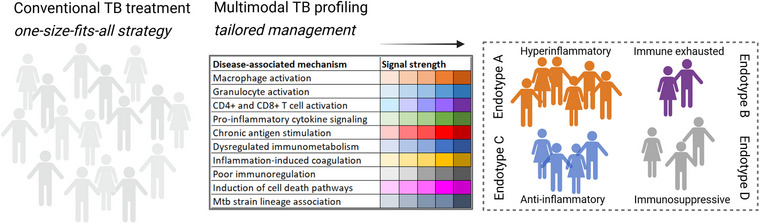
From uniform care to tailored insight: multimodal profiling in tuberculosis (TB). This figure illustrates the multimodal stratification of TB by integrating clinical phenotypes with immune endotypes. Clinical parameters, including symptom profiles, radiological findings, and treatment responsiveness, are combined with immunological mechanisms such as hyper‐ or hyporesponsiveness to capture the full spectrum of disease heterogeneity. Putative immune endotypes capture the underlying immune dysfunctions: hyperinflammatory (enhanced innate and adaptive responses), immune‐exhausted (chronic antigen exposure and up‐regulated checkpoint molecules), anti‐inflammatory (tissue‐remodeling and fibrosis), and immunosuppressed (induction of Treg cells and myeloid‐derived suppressor cells [MDSCs]). Together, these layers provide a framework for personalized disease characterization and targeted therapeutic strategies. By linking clinical presentation to underlying immune mechanisms, this approach enables refined patient stratification, identification of distinct disease trajectories, and guidance for host‐directed interventions.

Consistent with these results, another recent study using weighted correlation network analysis identified 17 transcriptional modules associated with TB lesions compared to non‐lesional tissue involving a gradient of immune‐related transcript abundance from the central core to the internal and external wall of the granuloma [117]. As such, patients with cavitary TB and more severe disease outcomes also presented an overabundance of immune inflammation‐related gene expression profiles, and downregulated tissue repair and metabolic modules [117]. It is likely that additional immune endotypes will be identified in the future by integrating clinical phenotypes and Mtb strain diversity with multi‐omics data and functional immune analyses of patient samples using multimodal profiling (Fig. 4).

#### Toward functional immune profiling in TB

##### Immune assessment: bridging clinical gaps in TB

**Fig. 5:**
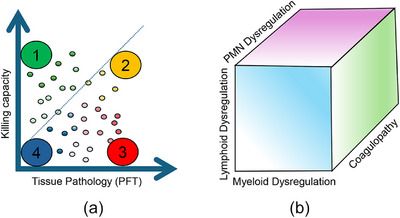
Framework for immune functional testing in tuberculosis (TB) care. The immune system must balance intracellular killing of Mycobacterium tuberculosis (Mtb) and prevention of immune‐mediated pathology and tissue damage. (a) To implement endotype‐specific host‐directed therapies (HDTs), clinicians must first assess both the host's capacity to eliminate Mtb and the extent of immune‐induced pathology. (b) If tissue pathology is present, identifying its underlying cause, whether driven by lymphocytes, monocytes, neutrophils, or coagulopathy, is essential for guiding targeted interventions.

Despite major technological advances and successful implementation in other medical fields, corresponding progress in infectious diseases, including assessment of immune responses in TB patients, remains largely unchanged. Current clinical practice lacks effective methods to evaluate the functional capacity of host immunity to contain or eliminate Mtb without causing collateral tissue damage. As such, clinicians need practical tools to assess two critical dimensions: (1) the host's ability to kill Mtb and (2) the extent of immune‐mediated tissue pathology (Fig. 5a). If tissue pathology is detected, then the cellular or functional origin of the immune dysregulation needs to be determined (Fig. 5b). As discussed, TB is characterized by a wide spectrum of immune perturbations, including T cell anergy, immune exhaustion, cytokine‐driven immunopathology, monocyte tolerance, hemophagocytic lymphohistiocytosis, neutrophil‐mediated tissue injury, natural killer cell deficiency, and immune‐induced coagulopathies. If systemic inflammation and tissue pathology are considered to promote TB disease, the next step would be to develop tests to specifically identify the dysregulated arm of the immune response. Although commercial assays are emerging to quantify viable Mtb in sputum, there are currently no standardized tests to measure immune‐mediated bacterial killing. In contrast, tissue and systemic inflammation can be evaluated using a combination of imaging, lung functional tests (e.g., spirometry), and simple blood‐based biomarkers such as CRP, ESR or immune‐inflammation markers or indexes. These tests need to continue to improve such that clinicians can determine the cellular source of inflammation, and their functional contributions, enabling the selection of the most appropriate HDT.

Overall, this complex interplay, in which host immunity can both control infection and drive pathology, underscores the limitations of a one‐size‐fits‐all approach to TB treatment. Instead, a practical framework using tests designed to assess immune killing capacity and tissue damage could enable stratified HDT, even in resource‐limited settings. Such an approach would mark a critical step toward personalized TB care.

##### Computational methods: a path toward immune endotyping in TB

Currently, TB patients cannot be stratified into therapeutic subgroups based on immune endotypes, as no tools for endotyping are available for clinical use. Although no standardized definition or workflow exists, endotyping is typically performed using unsupervised clustering of omics and non‐omics data, for example, clinical phenotyping data. Common data sources include clinical records, drug response, survival outcomes, experimental models, and molecular or immune profiles such as transcriptomics, proteomics, and genome‐wide association studies (GWAS). Classic examples of endotyping include asthma (Type 2‐high vs. Type 2‐low) [118], sepsis (three to four robust blood transcriptomic groups) [119], cancer (six immune subtypes across 10,000 tumors) [120], rheumatoid arthritis [121], IBD (IBD1/IBD2) and biomarkers such as OSM, TREM1 [122], and Type 2 diabetes (data‐driven clinical clusters) [123]. More recent studies applying advanced machine learning and cross‐dataset approaches involving gene set variation analysis‐based clustering identified eight SLE endotypes [101], whereas latent class analysis defined outcome‐linked subgroups in traumatic brain injury [124]. Likewise, Uniform Manifold Approximation and Projection for Dimension Reduction was used to stratify sepsis patients by transcriptomic signatures [125], whereas Bayesian non‐negative matrix factorization (NMF) revealed 11 obesity endotypes from GWAS of >2 million individuals [126].

A proposed pipeline using these examples: The process begins by clearly defining the clinical question and cohort, followed by assembling and preprocessing data, which may include both clinical variables and multi‐omics modalities. Unsupervised methods, such as clustering or multi‐omics integration, are then applied to uncover patient subgroups. These emerging endotypes are annotated with relevant biology through pathway, cell type, or microenvironment enrichment and subsequently validated in internal and external datasets, with outcomes and therapy responses used as benchmarks. For settings with limited data, novel strategies, such as network embedding, have been proposed. For example, rare autoimmune and autoinflammatory diseases were reclassified by mapping disease genes onto protein–protein interaction networks, identifying a highly connected “core module,” and grouping diseases based on their network proximity [127].

Endotyping offers a powerful framework to uncover hidden biological diversity among patients and guide personalized interventions. Integrating immune assessment with modern computational tools could also transform TB care, moving from reactive treatment to mechanism‐based precision medicine.

#### Tailored host‐directed therapies based on immune endotype profiling

##### Foundations for immune‐targeted interventions

Delineating a roadmap that incorporates immune endotyping in the clinical workflow will facilitate the decision of standard chemotherapy and appropriate HDT. Over two decades ago, Peter Barnes emphasized the need to tailor adjunct immunotherapies to individual patients, particularly those with difficult‐to‐treat MDR‐TB [128, 129]. He also noted that, during the early phase of treatment, the immune response plays a limited role in mycobacterial clearance compared to antibiotics, underscoring the importance of carefully evaluating the timing, dosage, and delivery route of biological agents [129]. Some immunotherapies—such as recombinant cytokines or cytokine inhibitors—as well as other forms of HDTs may be better suited for low‐dose administration after the intensive phase of anti‐TB treatment, supporting recovery and promoting complete cure. One example of a carefully calibrated intervention is the partial suppression of excessive TNF‐α levels in TB patients, without complete blockade, which enhanced IFN‐γ production and led to significant weight gain in TB patients [130].

Although the diverse approaches to HDTs have been thoroughly reviewed elsewhere [131, 132, 133], this section highlights selected examples illustrating how distinct immune endotypes can guide appropriate HDT strategies.

##### Strategies to modulate cytokine and chemokine signaling

Similar HDT strategies should be promoted and implemented to treat specific immune endotypes. For example, when excessive tissue pathology is linked to an overactive myeloid response, HDT with IL‐1 receptor antagonist (IL‐1RA) agents such as anakinra may be beneficial in combination with standard antibiotics. In a recent study, IL‐1RA therapy reduced bacterial burden and tissue damage in *Escherichia coli*–infected mice with acute cystitis by dampening inflammasome activation, immune cell infiltration, and inflammatory mediators [134]. Notably, anakinra also accelerated clearance of antibiotic‐resistant *E. coli*, underscoring IL‐1 blockade as a promising strategy to restore innate immune control and improve outcomes [134]. Conversely, IL‐1 signaling can enhance host resistance by inducing eicosanoids such as prostaglandin E2 (PGE2), which limit excessive Type I IFN and support Mtb control [135]. Accordingly, HDTs that elevate PGE2 levels have been shown to reduce TB progression in mice [135]. However, Mtb‐induced upregulation of PGE2 in myeloid cells may also promote expansion of FoxP3+ Treg that suppress effector T cell function and impair bacterial clearance [136]. These findings highlight the double‐edged nature of pro‐inflammatory cytokine signaling in TB, a complexity also observed with IL‐6 [37] and TNF‐α [137] pathways. To safely leverage cytokine antagonists, endotyping is essential for targeting the specific immune mechanisms driving disease progression and enhancing the precision of HDTs.

Similar to targeting pro‐inflammatory cytokines, chemokine or chemoattractant blockade could have a beneficial effect on immune cell composition and granuloma structure. Mtb disrupts normal immune cell trafficking to the infection site, undermining granuloma formation and promoting bacterial persistence [3]. Although MMPs play a key role in extracellular matrix degradation, these enzymes also act as potent immunomodulators capable of activating or inactivating cytokines and chemokines. For example, MMP‐2, MMP‐3, and MMP‐9 can process IL‐1β into an active and potent form at the site of infection [138], and MMP‐9 can also truncate IL‐8 (CXCL8) into a form with markedly enhanced neutrophil‐recruiting activity, creating a feed‐forward inflammatory loop [139]. Experimental models have shown that epithelial cells upregulated MMP‐9 expression in response to Mtb infection, enhancing macrophage recruitment, granuloma expansion, and mycobacterial growth [65]. As such, we have demonstrated that inhibition of MMP‐9 interferes with early granuloma formation and bacterial burden at the infection site [61]. Conversely, deficiency of CCL5 delayed IFN‐γ responses, reduced effector T cell recruitment, and impaired control of Mtb growth [140]. By distorting chemokine gradients, Mtb further misdirects immune cell trafficking into lesions, weakening host defenses and deepening immune dysfunction.

##### Strategies to limit hyperinflammation and coagulation to reduce TB‐related cardiovascular risk

Instead, if coagulopathy is the primary contributor to TB pathology, adjunct HDTs, such as aspirin, heparin, or statins, may offer greater benefit by inhibiting platelet aggregation and enhancing fibrinolytic activity, thereby mitigating inflammation‐driven thrombosis. Here, a randomized trial in Vietnam showed that adjunctive aspirin improved outcomes in patients with TB meningitis, likely by inhibiting pro‐thrombotic mediators and promoting the resolution of inflammation [141]. Although certain statins may reduce hypercoagulability and lower the risk of venous thromboembolism in patients [142], studies in mouse TB models have shown that statins also confer therapeutic benefits through mechanisms potentially independent of anticoagulation, including modulation of macrophage functions and inflammatory pathways [143, 144].

Systemic inflammation and thrombotic risk in TB patients can be mitigated through anti‐inflammatory therapies, including nonsteroidal anti‐inflammatory drugs, glucocorticoids, and anti‐platelet agents [31]. In preclinical studies, ibuprofen has been shown to improve survival, reduce bacterial load, and decrease the size and number of lung lesions in Mtb‐infected mice [145]. However, to minimize reliance on broad‐spectrum immunosuppressive drugs, more specific agents should be developed—for example, guided by assessments of neutrophil functions that correlate with the severity of lung pathology. Rather than broadly inhibiting neutrophil activity, selective blockade of neutrophil‐derived inflammatory mediators such as MMPs, calprotectin, or myeloperoxidase represents a promising strategy for developing more effective anti‐inflammatory HDTs for TB [23].

Several metabolic immune checkpoints, such as itaconate, also exhibit anti‐inflammatory properties and can modulate immune responses through diverse mechanisms, including epigenetic regulation [146]. By dampening excessive inflammation, these pathways may acutely reduce immune‐mediated pathology in TB. However, such treatment may also lead to sustained epigenetic‐driven immune suppression, which could potentially increase the risk of post‐TB cancers or recurrent infections.

##### Strategies to restore T cell function

In some cases, functional unresponsiveness or exhaustion of primarily the T cell response may represent a protective adaptation to limit immunopathology in TB [147]. However, if T cell dysfunction and low immune killing capacity persists months into conventional antibiotic therapy, further investigations and evaluation of adjunct treatment options are warranted. Immunotherapy with recombinant IL‐2 in human trials [148] and macaque studies [149] suggests that IL‐2 can break immune tolerance, expand effector T cell populations, and improve treatment outcomes in MDR‐TB. In mice, persistent stimulation with Mtb antigens also showed that IL‐2 restores T cell function by enhancing IFN‐γ production and reducing PD‐1 expression on antigen‐specific T cells [150]. In theory, this should restore antimicrobial activity in macrophages, prevent the development of tolerogenic dendritic cells, and enhance the activation and recruitment of cytotoxic T cells [151]. However, as with cytokine antagonists, recombinant cytokine therapies must be carefully tailored to patient‐specific immune endotypes to prevent unintended immune activation.

An alternative approach to follow up on persistent T cell dysfunction may involve screening for autoantibodies targeting Th1 or Th17 cytokines [152], which can indicate immune dysfunctions that are potentially treatable with the B cell‐depleting agent rituximab. For example, autoantibodies against IFN‐γ have been linked to chronic, disseminated, and treatment‐refractory infections caused by non‐tuberculous mycobacteria, which can be successfully managed with adjunct rituximab therapy [153, 154, 155]. Furthermore, metabolic reprogramming with metformin can reverse suboptimal T cell immunity involving increased mitochondrial dysfunction and inhibitory receptor expression, giving rise to an Mtb‐specific CD8+ T cell population with enhanced metabolic fitness [53].

##### Strategies to correct nutritional deficiencies

One of the most promising HDT strategies in TB involves correcting nutritional deficiencies that contribute to immune dysfunction. Targeted interventions with cod liver oil (rich in carotenoids, vitamin A, vitamin D_3_, and anti‐inflammatory omega‐3 fatty acids) supplementation and dietary support to prevent cachexia and undernutrition can potentially help restore immune function. A cluster‐randomized controlled trial in India recently demonstrated that daily supplementation with food rations and micronutrients led to a substantial reduction in TB incidence, ranging from 39% to 48%, among household contacts over a 2‐year follow‐up period [156]. Moreover, arginine may serve as a beneficial adjuvant therapy in patients with active TB, primarily by enhancing the production of nitric oxide, a molecule with both bactericidal and immunomodulatory properties [157]. Likewise, daily adjunct HDT using nutritional supplementation with vitamin D_3_ and the butyrate derivative phenylbutyrate (PBA) has been shown to reduce both sputum bacterial load and clinical TB score, particularly in patients with moderate to severe TB [84]. Patients exhibited a bimodal response to adjunct therapy, with a subset showing markedly better outcomes [84]. This suggests that individual benefit may depend not only on disease severity but also on underlying immune endotypes that shape responsiveness to host‐directed interventions. Improved clinical outcomes were also linked to enhanced immune function in both myeloid and T cell compartments [158], including reduced inflammatory cytokines and ER‐stress [159]. In vitro studies of vitamin D_3_ and PBA further demonstrated enhanced macrophage effector functions such as induction of autophagy and antimicrobial peptides, which have additive effects with antibiotics and could aid killing of MDR‐TB [160]. Collectively, these findings support the concept that targeted nutritional interventions can restore immune competence, accelerate recovery, and potentially shorten the duration of TB treatment.

## Conclusions

Despite decades of standardized antibiotic regimens, TB care still overlooks the profound immune variability that shapes clinical phenotypes. Precision medicine offers a path forward by tailoring therapies to patient subgroups that share clinical features but diverge in underlying immune mechanisms.

TB spans a spectrum—from latent infection to subclinical/incipient or mild–moderate–severe, non‐cavitary, cavitary, or miliary disease—that arises from distinct, and often competing, immune programs. Latent TB reflects durable Th1‐mediated containment without sterilizing cure; subclinical TB shows early IFN‐driven innate signatures with limited systemic inflammation. Progression to clinically active disease brings intensified inflammation, metabolic reprogramming, shift in the Th1/Th2 balance, cytolytic T cell dysfunction, and tissue remodeling and immune exhaustion in moderate–severe cases. Non‐cavitary disease often indicates partial Th1‐dominated control and lower relapse risk, whereas cavitary disease reflects uncontrolled, MMP‐driven pathology with high bacillary burden and transmission risk. Miliary TB signals failed granuloma control in hosts with impaired cell‐mediated immunity. These divergent trajectories explain why a one‐size‐fits‐all approach cannot fully address morbidity, treatment failure, or post‐TB sequelae.

Moving forward, TB endotypes must be defined at cellular and functional levels by integrating deep‐immune profiling that combines transcriptomic, proteomic, metabolic, lipidomic, and epigenomic data with clinical/radiological scoring and bacterial strain diversity (Table 1 and Fig. 4). A practical precision framework combines multimodal modeling using: (1) clinical and radiological phenotyping to assess disease extent and severity, (2) immune endotyping to identify myeloid, lymphoid, or granulocytic dysregulation, such as IFN‐high inflammation, coagulation disturbances, Th2/fibrotic remodeling, exhaustion or immunosuppression, and impaired effector function, and (3) metabolic and tissue‐remodeling markers to guide tailored HDTs alongside optimized antibiotics.

Illustrative strategies include targeted control of hyperinflammation (e.g., MMP inhibition, IL‐1/IL‐6 modulation), anti‐thrombo‐inflammatory interventions (aspirin/statins) when coagulopathy predominates, restoration of T cell function (judicious cytokine support, metabolic reprogramming), and nutritional interventions to reverse chronic malnutrition and immunodeficiency. Early risk stratification in subclinical disease can trigger preventive therapy before irreversible damage occurs. In non‐cavitary disease, the focus is supporting containment without over‐suppression, whereas in cavitary, high‐burden disease, priorities include limiting tissue destruction and improving drug penetration. Embedding this phenotype‐plus‐endotype framework in clinical trials and practice promises shorter, safer, and more effective regimens aligned with each patient's dominant immune and pathological drivers rather than the pathogen alone.

## Conflict of interest statement

The authors declare no conflicts of interest.

## Funding information

Swedish Heart and Lung Foundation (HLF) and King Oscar II Jubilee Foundation (2025‐0556 to S.B.), the Swedish Research Council (VR) (2022‐00970, 2022‐03174, and 2024‐06106 to S.B.), the Center for Innovative Medicine (CIMED to S.B.), ALF Region Stockholm (S.B.), the Foundation to Prevent Antibiotic Resistance (PAR to S.B.), and the Scandinavian Society of Antimicrobial Chemotherapy (SSAC to S.B.)

## Disclosure

The funders had no role in the preparation or content of this manuscript.
